# β-sitosterol attenuates high- fat diet-induced hepatic steatosis in rats by modulating lipid metabolism, inflammation and ER stress pathway

**DOI:** 10.1186/s40360-023-00671-0

**Published:** 2023-05-12

**Authors:** Omayma AR Abo-Zaid, Fatma SM Moawed, Effet Soliman Ismail, Mostafa A. Farrag

**Affiliations:** 1 Molecular Biology Department, Faculty of Vet. Med, Benha University, Banha, Egypt; 2 Health Radiation Research, National Center for Radiation Research and Technology, Egyptian Atomic Energy Authority, Cairo, Egypt; 3 Radiation Biology, National Center for Radiation Research and Technology, Egyptian Atomic Energy Authority, Cairo, Egypt

**Keywords:** NAFLD, β-sitosterol, Endoplasmic reticulum stress, PPAR-α, SREBP-1c, CPT-1

## Abstract

Nonalcoholic fatty liver disease (NAFLD) is the most prevalent chronic hepatic disorder. The naturally occurring phytosterol; β-sitosterol has antiobesogenic and anti-diabetic properties. The purpose of this study was to explore the role of β-sitosterol in preventing hepatic steatosis induced by a high-fat diet (HFD) in rats. In the current study, to induce NAFLD in the female Wister rats, an HFD was administered to them for 8 weeks. The pathogenic severity of steatosis in rats receiving an HFD diet was dramatically decreased by oral administration of β-sitosterol. After administering β-sitosterol to HFD-induced steatosis for three weeks, several oxidative stress-related markers were then assessed. We showed that β-sitosterol reduced steatosis and the serum levels of triglycerides, transaminases (ALT and AST) and inflammatory markers (IL-1β and iNOS) compared to HFD-fed rats. Additionally, β-sitosterol reduced endoplasmic reticulum stress by preventing the overexpression of inositol-requiring enzyme-1 (IRE-1α), X-box binding protein 1(sXBP1) and C/EBP homologous protein (CHOP) genes which, showing a function in the homeostatic regulation of protein folding. Also, it was found that the expression of the lipogenic factors; peroxisome proliferator-activated receptor (PPAR-α), sterol regulatory element binding protein (SREBP-1c) and carnitine palmitoyltransferase-1(CPT-1), which are involved in the regulation of the fatty acid oxidation process, may be regulated by β-sitosterol. It can be concluded that β-sitosterol may prevent NAFLD by reducing oxidative stress, endoplasmic reticulum stress and inflammatory responses, which supports the possibility of using β-sitosterol as an alternative therapy for NAFLD. Together, β-sitosterol may be an option for NAFLD prevention.

## Introduction

Nonalcoholic fatty liver disease (NAFLD) is a chronic liver condition that affects people who drink infrequently or not at all and do not have viral hepatitis. Its hallmark is the accumulation of extra lipids inside hepatocytes. Simple steatosis, a benign disease, is at the mild end of the NAFLD spectrum. Nonalcoholic steatohepatitis (NASH), however, can proceed to cirrhosis and liver failure [1]. NAFLD is regarded as a hepatic manifestation of the metabolic syndrome and typically develops in conjunction with diseases linked to insulin resistance [2]. Additionally, the prevalence of NAFLD has dramatically grown along with the global prevalence of obesity [3]. Over the past 20–30 years, prevalence rates of NAFLD in Western and Asian nations have been between 20 and 30% and 25% of the general population, respectively [4]. NAFLD is one of the most prevalent liver disorders at the moment, and its clinical burden is anticipated to increase in the coming years.

Protein folding, lipid and sterol synthesis, and intracellular Ca2 + storage are all mediated by the endoplasmic reticulum (ER), which is essential for the synthesis of membrane and secreted proteins. The accumulation of unfolded protein in the ER lumen, however, is caused by pathogenic events that disturb ER homeostasis and cause ER stress. Typically, the unfolded protein response (UPR) upregulates protein chaperones, inhibits protein translation, and removes unfolded proteins to help cells survive early stress (UPR). However, protracted ER stress can result in cell death and numerous disorders, including ischemia/reperfusion damage, heart disease, and diabetes. According to recent research, metabolic disorders including obesity and diabetes are associated with hepatic ER stress. In non-alcoholic fatty liver disease, ER stress helps to cause hepatic steatosis and insulin resistance (NAFLD). ER, stress is reportedly a significant pathogenic element in this disease process. To treat NAFLD, a substance that may reduce ER stress may be a useful therapeutic choice [5].

A successful medicinal intervention strategy for NAFLD does not yet exist. Due to the positive outcomes, they have demonstrated, natural substances are considered effective therapy options to slow the progression of NAFLD [6, 7]. One of the most widespread phytosterols is β-sitosterol, which is considered an anti-obesogenic, anti-inflammatory, and anti-diabetic agent. According to research, β-sitosterol’s antioxidant properties and capacity to promote pancreatic β-cell regeneration are what cause it to have anti-diabetic benefits [8, 9]. Low-density lipoprotein (LDL) in hypercholesterolemic individuals has been successfully treated in clinical research by the use of phytosterols [10]. In a rat model, the earlier research by Gumede et al. [11, 12] supported the protective effect of β-sitosterol against metabolic dysfunction and NAFLD. However, it is still unclear how β-sitosterol works to treat NAFLD by lowering hepatic steatosis. Therefore, the purpose of this study was to determine if supplementing with β-sitosterol in the NAFLD rat model will decrease hepatic steatosis by reducing ER stress and controlling the rat’s fatty acid oxidation process.

## Materials and methods

### Chemicals

β-sitosterol was provided by BASF China. All the reagents and chemicals used for this research were acquired from Sigma Chemical Company, St. Louis, MO, USA.

### Experimental animals and treatment

#### Animals

From the Nile Company for pharmaceuticals thirty female Wister rats (120 ± 10 g) were obtained.

Thirty female Wister rats (120 ± 10 g) were purchased from the Nile Company for pharmaceuticals. Six animals were housed in an air-conditioned (25 ± 2 °C) animal housing with free access to water and a normal laboratory feed (El-Nasr Co. Cairo, Egypt) and exposed to a 12:12-h light-dark cycle. The experimental animals were treated in accordance with the guidelines of Ethics by the Guide for the Care and Use of Laboratory Animals 1997, published by Clark et al. 23 and the recommendations of the animal care committee of the National Center for Radiation Research & Technology (NCRRT), Cairo, Egypt. Moreover, the present study was approved by the Institutional Animal Care and Use Committee Research Ethic Board (BUFVTM 06-04-022).

### Experimental design

After a week of acclimation, some of the animals were fed conventional chow, while others were administered a high-fat diet (HFD) for 8 weeks. The HFD received from El-Nasr Co. (Cairo, Egypt) had 50% carbohydrates/starch, 27% fat, 10% protein, 10% sucrose, 1.5% fiber, and 1.5% vitamins. The animals were divided into three groups of ten each:

**Group 1** (**control**): Rats receiving standard chow.

**Group 2** (**HFD**): Rats were fed with HFD.

**Group 3** (**HFD + βsito**): HFD-fed rats received β-sitosterol orally by gavage at daily doses of 20 mg/kg B.W for 30 days [13].

Before and after the administration of the medications, the rats’ body weights were continuously monitored throughout the experiment. By retro-orbital bleeding into tubes, blood samples were obtained. In preparation for further analysis, serum samples were separated, split into aliquots, and kept at ‒80°C. After cervical dislocation was used to sacrifice all of the rats, the liver was removed, cleaned with saline, dried on filter paper, weighed, and then kept at temperatures up to 80° C for further mRNA extraction.

### Biochemical analyses

Peroxisome proliferator-activated receptor gamma coactivator-1a (PGC-1a), Carnitine palmitoyltransferase 1 A (CPT1A), and PPAR-α levels in the liver were measured using an ELISA kit from My Biosource Inc., San Diego, California, USA (Cat No. MBS2504779), (Cat Nos. MBS2020426), and (Cat No. MBS1600202), respectively Interleukin-1 (IL-1) and inducible nitric oxide synthase (iNOS) levels in liver tissue were estimated using ELISA kits from My Biosource Inc., San Diego, California, USA (Cat Nos. MBS2125216 and MBS263618, respectively). Using an ELISA kit purchased from My Biosource Inc., San Diego, California, USA, (Cat Nos. MBS2125216 and MBS263618), the levels of interleukin-1 (IL-1) and inducible nitric oxide synthase (iNOS) were estimated in liver tissue. A commercial kit from Cairo, Egypt’s Biodiagnostic Company was used to assess the serum triglycerides (TG) and cholesterol levels. Malondialdehyde (MDA), a sign of lipid peroxidation, was measured in liver homogenates using a method that has been described [14]. PON-1 and ARE activities in liver tissue were assessed spectrophotometrically according to the procedure outlined by Gan et al. [15]. Aspartate transaminase (AST, EC 2.6.1.1) and alanine transaminase (ALT, EC 2.6.1.2) serum activities were determined according to the instructions of commercial kinetic assay kits acquired from Spectrum Diagnostic Company, Cairo, Egypt.

### Detection of gene expression by real-time quantitative polymerase chain reaction (PCR)

#### Isolation of RNA and reverse transcription

Bcl-2-associated X protein (Bax), B-cell lymphoma 2 (Bcl-2), inositol-requiring enzyme-1 (IRE-1α), C/EBP-homologous protein (CHOP), X-box binding protein 1 (sXBP1), glucose-regulated protein 78 (GRP78), and sterol regulatory element binding protein (SREBP-1c) mRNA expression levels were determined and investigated (see Table-1 for primer sequences used in these investigations). From 30 mg of liver tissues, total RNA was extracted using the TRIzol reagent (Life Technologies, USA) according to the manufacturer’s instructions. To ensure the validity of the RNA, ethidium bromide staining and agarose gel electrophoresis (1% each) were utilized. Reverse transcriptase (Invitrogen) was used according to the manufacturer’s instructions to produce the first strand complementary DNA (cDNA) using 1 µg of total RNA as the template. RT-PCRs were carried out in a thermal cycler stage one plus using the Sequence Detection Program (PE Biosystems, CA) (Applied Biosystems, USA). 2X SYBR Green PCR Master Mix (Applied Biosystems), 900 nM of each primer, and 2 µL of cDNA were all combined into a reaction mixture with a total volume of 25 µL. The PCR thermal cycling settings included the first step at 95 °C for 5 min, 40 cycles at 95 °C for 20 s, 60 °C for 30 s, and 72 °C for the 20s. A curve analysis was performed following the reaction. The results were standardized using the β-actin gene that was amplified in each round of PCR tests. Using the comparative Ct approach outlined by Livak and Schmittgen [16], the relative expression of the target mRNA was assessed.

**Table 1:** Primer sequences used for RT-PCR

Primer	Sequence
**Bax**	Forward: 5ʹ- ACACCTGAGCTGACCTTG -ʹ3 Reverse: 5ʹ- AGCCCATGATGGTTCTGATC -ʹ3
**Bcl2**	Forward:5′- ATCGCTCTGTGGATGACTGAGTAC − 3’ Reverse: 5′- AGAGACAGCCAGGAGAAATCAAAC − 3’
***IRE1α***	Forward:5ʹ- TTGACTATGCAGCCTCACTTC -ʹ3 Reverse: 5ʹ- AGTTACCACCAGTCCATCGC -ʹ3
**CHOP**	Forward: 5′- ACCACCACACCTGAAAGCAG -ʹ3 Reverse: 5′- AGCTGGACACTGTCTCAAAG -ʹ3
**XBP1**	Forward:5ʹ- TGAAGCTTTGCGTAGTCTGGAGCTATGG -ʹ3 Reverse: 5ʹ- TGCTCGAGATTGGATCATTCCTTAGACA -ʹ3
**GRP78**	Forward: 5ʹ- AACCCAGATGAGGCTGTAGCATA -ʹ3 Reverse:5ʹ - CACAGTGTTCCTCGGAATCAGTT -ʹ3
**SERBP1c**	Forward:5ʹ- GGCCCTGTGTGTACTGGTCT -ʹ3 Reverse: 5ʹ- AGCATCAGAGGGAGTGAGGA -ʹ3
**B-actin**	Forward: 5ʹ- AAGTCCCTCACCCTCCCAAAAG -ʹ3 Reverse:5ʹ - AAGCAATGCTGTCACCTTCCC -ʹ3

### Histopathological analysis

For hematoxylin-eosin (H&E) staining, the livers were processed, embedded in paraffin, and preserved in 10% buffered formalin. Using a light microscope, the microscope slides were examined (Olympus Soft Imaging Solutions GmbH, Munster, Germany).

### Statistical analysis

The statistical software SPSS (Statistical Program for Social Science) version 20.0 was used to do statistical analysis on the data and perform tests of significance. A one-way ANOVA test was used, followed by a post hoc test for multiple comparisons. All data are reported as a mean of 8 values, and P < 0.05 is used to determine if the SE and difference between means are significant.

## Results

### Effects of β-sitosterol treatment on metabolic parameters

After an 11-week study period, all groups’ body weights, liver weight, adipose tissue weight and serum biochemical markers were assessed (Table 2). In comparison to the control group, HFD rats had substantially greater body weight, liver weight, adipose tissue weight, cholesterol, triglyceride (TG), aspartate aminotransferase (AST), and alanine aminotransferase (ALT) levels. However, body weight, liver weight, adipose tissue weight, the activities of liver enzymes (ALT and AST) and lipid indices (cholesterol and TG) were dramatically reduced in the rats given HFD for three weeks after the administration of β-sitosterol in comparison to the HFD group.

**Table 2:** Effects of β-sitosterol on metabolic parameters (ALT, AST, cholesterol, TG and body weight) in serum of rats groups

Groups	Control	HFD	βSito + HFD
ALT U/ml	8.6 ± 0.42^b^	17.8 ± 1.00^a^	10.6 ± 0.84^b^
AST U/ml	7.0 ± 0.44^b^	17.2 ± 1.70^a^	10.6 ± 0.42^ab^
Cholesterol (mg/ dl)	62.0 ± 0.89^b^	77.60 ± 1.46^a^	64.0 ± 1.26^b^
TG (mg/ dl)	79.5 ± 0.56^b^	95.8 ± 3.70^a^	84.00 ± 2.50^a^
Body weight (Grams)	164.6 ± 2.01^bc^	203 ± 2.00^ac^	183.5 ± 2.01^ab^
Liver weight (Grams)	5.3 ± 0.17^bc^	6.8 ± 0.17^ac^	6.0 ± 0.11^ab^
Adipose tissue weight (Grams)	4.8 ± 0.23^bc^	9.3 ± 0.11^ac^	6.4 ± 0.23^ab^

Data were expressed as Mean ± S.E. ^a^*P* < 0.05 versus the control group, ^b^*P* < 0.05 versus the HFD group, ^C^*P* < 0.05 versus the HFD + βSito group

### Effects of β-sitosterol treatment on hepatic expressions of lipid metabolism

**Fig. 1:**
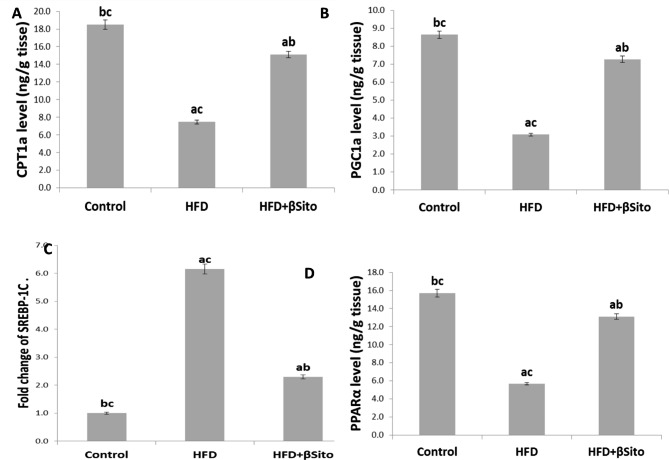
Hepatic levels of lipogenic and mitochondrial biogenesis factors in HFD-fed rats. **(A)** CPT1a level **(B**) PGC-1a level (**C**) SREBP-1c gene expression **(D)** PPARα level in all groups Data were expressed as Mean ± S.E. ^a^*P* < 0.05 versus control group, ^b^*P* < 0.05 versus HFD group, ^C^*P* < 0.05 versus HFD + βSito group

Hepatic levels of lipogenic and fat oxidation enzymes were measured to ascertain if β-sitosterol had an impact on the enzymes responsible for hepatic lipid metabolism. As shown in Fig. 1, when compared to the control group, it was shown that the HFD-fed rats had significantly lower levels of lipogenic factors such as PGC-1a, PPARα, and CPT-1a and higher levels of the gene SREBP-1c. The levels of PGC-1a, PPARα, and CPT-1 as well as SREBP-1c gene expression were, however, substantially altered in the βSito + HFD group compared to the HFD group (P < 0.05).

### Effects of β-sitosterol treatment on hepatic expression of inflammatory markers

**Fig. 2:**
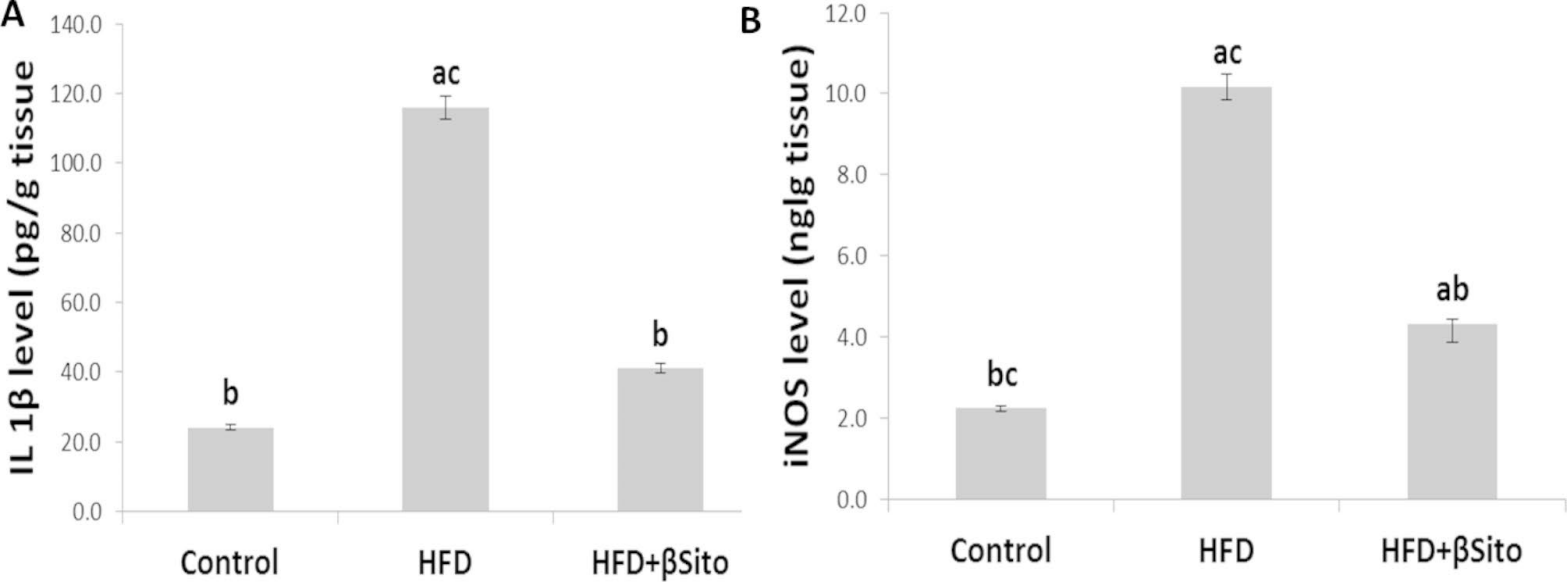
Hepatic levels of inflammatory markers **(A)** IL-1β and **(B)** iNOS in all groups. Data are expressed as mean ± SE. ^a^*P* < 0.05 versus control group, ^b^*P* < 0.05 versus HFD group, ^c^*P* < 0.05 versus HFD + βSito group

The levels of hepatic pro-inflammatory markers (IL-1β and iNOS) were evaluated to determine the effect of β-Sitosterol treatment on liver inflammation. Hepatic levels of IL-1β and iNOS were significantly increased in the HFD group, and this increase was abolished in the HFD + βSito group (*P* < 0.05) (Fig. 2).

### Effects of β-sitosterol treatment on hepatic expression of oxidative stress markers

**Fig. 3:**
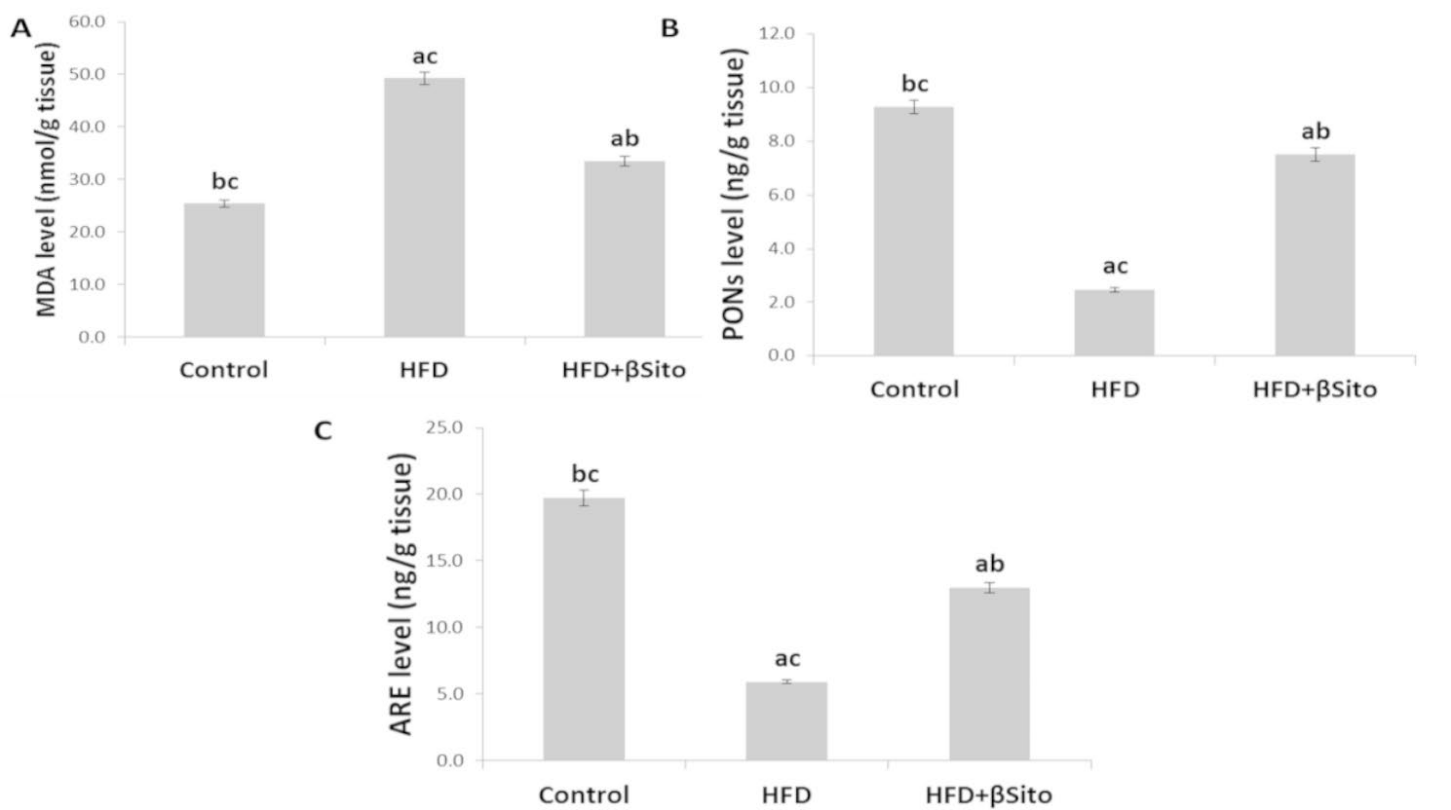
Hepatic oxidative stress biomarkers **(A)** MDA level, **(B)** PONs activity, **(C)** ARE activity in all groups. Data were expressed as Mean ± S.E. ^a^*P* < 0.05 versus the control group, ^b^*P* < 0.05 versus the HFD group, ^C^*P* < 0.05 versus the HFD + βSito group

To test the antioxidant effect of β-Sitosterol in the liver of HFD-fed rats, we first measured selected parameters associated with oxidative stress in HFD and HFD + βSito as compared to control rats. One of the primary events during oxidative stress is the peroxidative damage to membrane lipids, which can be determined by their degradation product MDA. Indeed, an increased level of MDA was measured in HFD compared to control rats. The oxidative condition of the liver of HFD rats was accompanied by reduced activity of ROS-detoxifying enzymes such as ARE and PONs whose activities were all reduced in HFD as compared to control rats. Thereafter, we determined whether the hepatic oxidative stress of HFD rats could be reduced by β-Sitosterol treatment. As result, the MDA level and activities of ARE and PONs were modulated in the HFD + βSito group compared to the HFD group (Fig. 3).

### Effects of β-sitosterol treatment on hepatic expression of endoplasmic reticulum (ER) stress markers

**Fig. 4:**
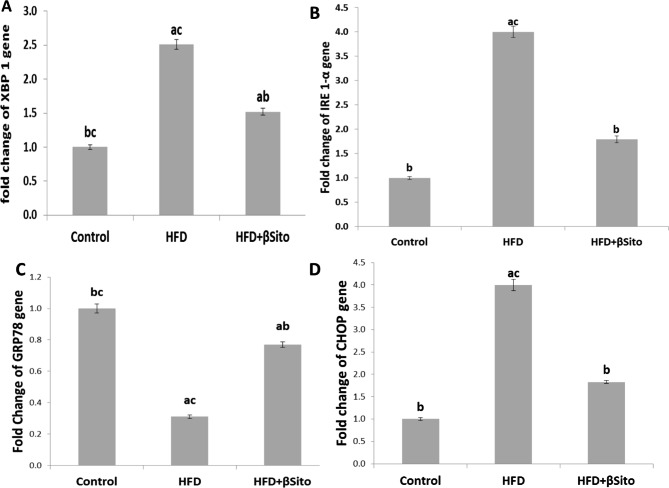
Hepatic ER stress pathway. **(A)** XBP-1, **(B)** IRE1α, **(C)** GRP78, **(D)** CHOP gene expression in all groups. Data were expressed as Mean ± S.E. ^a^*P* < 0.05 versus the control group, ^b^*P* < 0.05 versus the HFD group, ^C^*P* < 0.05 versus the HFD + βSito group

It is generally known that ER stress activates the unfolded protein response (UPR), which works to restore the normal ER homeostatic state within the cell. To determine whether β-Sitosterol can influence HFD-induced ER stress, the gene expression of GRP78, IRE1α, XBP1, and CHOP was measured in the livers of all groups of rats. Compared to the control group, the HFD group’s expression of IRE1α, XBP1, and CHOP was significantly upregulated, and this upregulation was linked to a decrease in the level of GRP78 gene transcript. Notably, the expression of each of these genes was altered in the HFD + βSito group compared to the HFD group, indicating that β-Sitosterol can effectively reverse the ER stress caused by the HFD in the rat liver (Fig. 4).

### Effects of β-sitosterol treatment on hepatic expression of ER stress-induced apoptosis

**Fig. 5:**
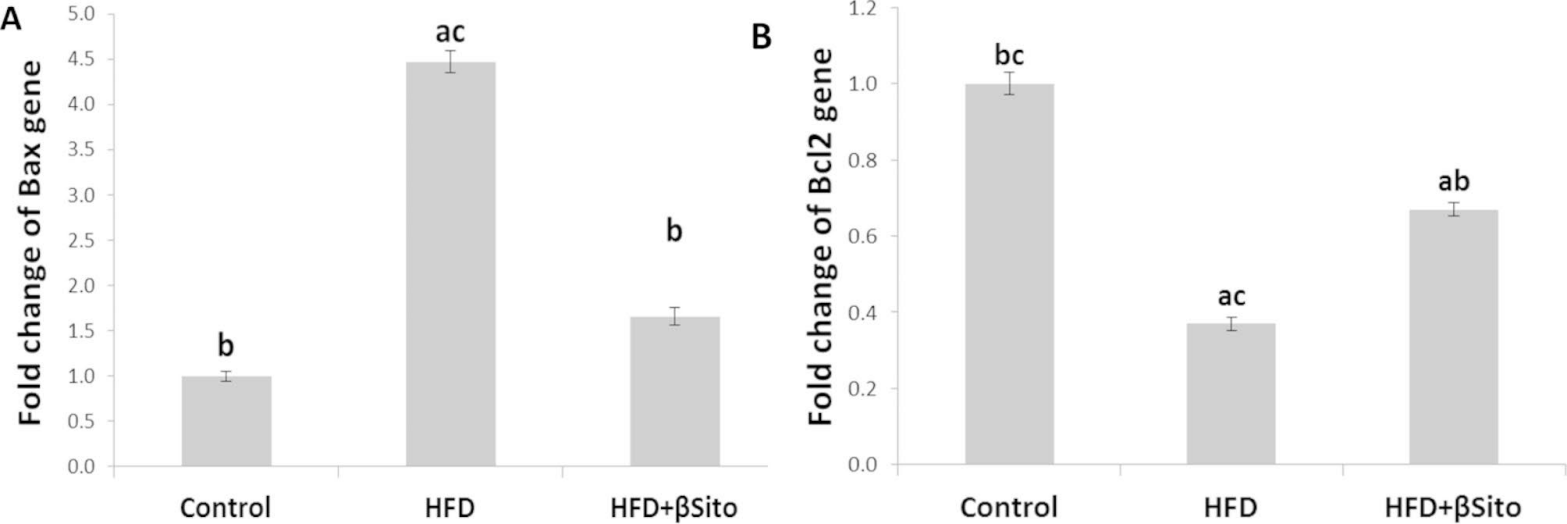
Hepatic gene expression of key marker for apoptosis **(A)** Bax and **(B)** Bcl-2 gene expression in all groups. The band intensity of the proteins was determined using an imaging densitometer, and the relative levels of each protein were calculated based on the intensity of actin protein as an endogenous control. Data represent the means ± SD from three replicates. *P

By activating the prolonged UPR, apoptosis might be induced. A change in the gene expression level of apoptotic proteins was examined in rats fed with HFD + βSito to determine if the ameliorative effect of β-Sitosterol for ER stress may be followed by the elimination of the ER stress-induced apoptosis. After consuming HFD, it was found that the liver tissue of the HFD group showed a significant elevation in the gene expression of Bax together with a downregulation in the gene expression of BCL2. However, there was a significant alteration in the expression of BCL2 and Bax mRNA in the HFD + βSito treatment group when compared to the HFD group. These findings imply that the improvement of the ER stress-induced apoptosis may be strongly associated with the suppression impact of β-Sitosterol on the ER stress (Fig. 5).

### Histopathological finding

**Fig. 6:**
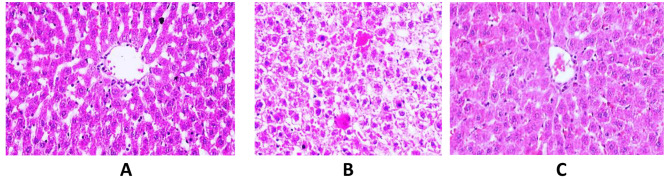
Photomicrographs of liver tissue section showing: **(A)** normal histological structure of hepatic lobules and organization of hepatic cords (Control group) **(B)** liver tissue section had ballooning hepatocyte degeneration together with narrowing hepatic sinusoids. There were a few intracellular fat droplets and hyperplasia of kuffper cells (grade III) (HFD fed group) **(C)** liver tissue had hyperplasia of kuffper cells, mild hepatocyte enlargement, and granularity in its cytoplasm (grade I) (HFD + βSito group) (H&EX400)

Liver tissue section of control group showed normal histological structure of hepatic lobules and organization of hepatic cords with prominent central hepatic vein. Polygonal hepatic cells were joined to one another in anastomosing plates, with borders that face either the sinusoids or adjacent hepatocytes (grade 0) (Fig. 6A). On the other hand, the HFD group’s liver tissue section had ballooning hepatocyte degeneration together with narrowing hepatic sinusoids. There were a few intracellular fat droplets and hyperplasia of kuffper cells (grade III) Fig. (6B). On the other hand, the animal group (HFD + βSito) had hyperplasia of kuffper cells, mild hepatocyte enlargement, and granularity in its cytoplasm (grade I) (Fig. 6C).

## Discussion

In the current work, a rat model of NAFLD and hepatic steatosis was established by a diet high in saturated fats, which was proved by histopathological analysis. Previous research has demonstrated that people with NAFLD had mild to moderately increased blood levels of AST and ALT. Rats in the HFD group had higher AST and ALT activity than rats in the control group, which showed that the NAFLD model had been successfully established. Additionally, the findings demonstrated that the HFD group’s blood cholesterol and TG values were considerably higher than those of the control group, pointing to a change in lipid metabolism. One of the major causes of hepatic steatosis is the increased absorption of fatty acids from plasma [17]. Lower fatty acid β-oxidation is another factor contributing to steatosis. In the current study, levels of CPT1a, PPARα (a crucial regulator of oxidation), and SREBP-1c (a lipogenic factor) were assessed in the liver tissue of HFD-fed rats and HFD rats treated with -sitosterol.

It was reported that the liver’s fatty acid oxidation processes and lipogenesis are closely related. Malonyl-CoA levels rise as a result of de novo lipogenesis, which also prevents the CPT-1 enzyme from mediating the entrance of long-chain acyl-CoA coenzymes into the mitochondria and the start of fatty acid breakdown *via* β-oxidation [18]. Since it controls mitochondrial beta-oxidation enzymes, PPARα, a member of the superfamily of ligand-activated nuclear hormone receptors, is also connected to hepatic lipid metabolism. According to the current study, rats fed an HFD had significantly lower levels of PPARα and CPT-1, which contributed to the pathogenesis of fatty liver. These findings were in line with other studies that demonstrated that excessive HFD in rats decreased the expression of the PPAR in the liver, which in turn inhibited the fatty acid oxidation enzymes and suppressed the export of hepatic lipids [19].

A plant sterol called β-sitosterol can be found in grains and vegetable oils. They are structurally similar to cholesterol but absorb at much lower rates, lowering blood cholesterol levels by partially preventing cholesterol absorption in the digestive tract [20]. The results of the current investigation showed that β-sitosterol decreased serum TG and cholesterol levels. Additionally, histological analysis revealed that the use of β-sitosterol dramatically reduced liver steatosis. Lower activities of AST and ALT showed that liver function had improved. However, in the HFD + βSito group compared to the HFD group, treatment with β-sitosterol resulted in a considerable increase in β-oxidation factors (CPT1a and PPARα) levels as well as a downregulation of the gene expression of a lipogenic factor (SREBP1C). This shows that treatment with β-sitosterol might reduce HFD-induced lipogenesis by downregulating the expression of lipogenic genes and upregulating the expression of lipid oxidation genes. These findings imply that β-sitosterol, which inhibits the absorption of cholesterol, may have positive effects on the development and progression of NAFLD.

A disruption in lipid metabolism may result in an oxidative stress state, which may subsequently lead to further structural and functional defects [21]. This study demonstrated that MDA, one of the lipid peroxidation’s byproducts, rose appropriately in HFD rats. According to the findings, oxidative stress has been greatly increased. The treatment of β-sitosterol in our study reduced the level of MDA. PONs and ARE activity, two enzymes that protect against oxidative stress, were increased by β-sitosterol.

Inflammation, fibrosis, and pathological angiogenesis are all consequences of lipotoxicity, which results from an excessive buildup of lipids in hepatocytes [22]. Reactive oxygen species, oxidative stress, and the activation of proinflammatory markers like IL-1β and iNOS all contribute to inflammatory reactions. According to the current work, it was shown that rats given an HFD had significantly higher levels of IL-1β [23] and iNOS activity, [24], which may be attributed to the induction of pro-inflammatory cytokines [25]. Additionally, it was shown that IL-1β, which is extensively expressed in the many subpopulations of liver cells, has a significant role in liver illness, including hepatic steatosis, inflammation, and fibrosis. Additionally, IL-1β may encourage hepatic steatosis by promoting the accumulation of triglycerides and cholesterol in primary liver hepatocytes as well as the development of lipid droplets [24]. However, supplementation of β-sitosterol to rats fed on HFD significantly attenuated the increase in the inflammatory markers (IL-1β and iNOS), which is agree with the finding of Plat et al., (2014), suggesting an anti-inflammatory potential of β-sitosterol in NAFLD [26].

Inflammation and hepatic steatosis can result from ER homeostasis disruption [27]. UPR activation and ER stress are brought on by disruptions in the processes of protein folding or modification [28]. The results of the current study showed that ingestion of HFD enhanced the activation of the CHOP, IRE1α, and XBP-1 pathways and triggered the hepatic ER stress pathway. Through the activation of ER stress, prior studies have shown that HFD feeding caused fat formation in primary hepatocytes [29, 30]. The expression of lipogenic genes in the liver is increased by XBP-1 and IRE1α activation-induced hepatic insulin resistance [31]. The control of hepatic very low-density lipoprotein assembly and secretion is also significantly influenced by the IRE1-XBP-1 pathway [32]. Also, previous work has shown that when ER stress is generated, the deletion of IRE1 in the liver causes the development of severe hepatic steatosis. In our study, we showed that ER stress activation, induced by HFD, was significantly reduced by β-sitosterol, as suggested by the lower expression of CHOP, IRE1 and XBP-1 in the HFD + βSito group versus the HFD group of rats. We might hypothesize that the β-sitosterol-induced anti-steatotic action would lower the pressure brought on by lipid deposition, hence reducing ER stress.

A cascade of ER stress, ROS generation, and inflammation are all linked together in the apoptotic signaling network. Apoptosis is further enhanced in the etiology of NAFLD by ongoing and severe ER stress [33]. To keep the ER in homeostasis, the apoptosis caused by ER stress should be inhibited accompanied by activation of UPR response. Therefore, other elements that trigger apoptosis are triggered if high ER stress continues. Additional well-known indicators linked to ER stress-mediated apoptosis include CHOP, Bax, and BCl2. According to reports, CHOP is a crucial indicator of ER stress-mediated apoptosis. In ER stress, CHOP has shown a reduction in apoptosis [34]. To treat NAFLD patients, it may be possible to target CHOP, Bax, and Bcl2. Similar to previous reports [35]; we also found that expression levels of CHOP and Bax were efficiently decreased by β-sitosterol in HFD-fed rats. These results suggest that β-sitosterol can reduce apoptosis by inhibiting ER stress.

In conclusion, β-sitosterol ameliorates HFD-induced hepatic steatosis through the modulation of hepatic lipid metabolism, pro-inflammatory markers, and the ER stress pathway. Therefore, β-sitosterol may have a direct role in the treatment of NAFLD. Additionally, β-sitosterol might be a promising lead compound for future drug development.

## Data Availability

The corresponding author (Fatma S.M. Moawed, fatmasearch5@yahoo.com) will provide the datasets produced and/or analyzed during the current work upon reasonable request.

## Declarations

### Permission for reproduced images

There is no imges are applicable in the current study.
